# The impact of yeast-encapsulated orange oil in *Aedes aegypti* oviposition

**DOI:** 10.1371/journal.pone.0301816

**Published:** 2024-05-14

**Authors:** Bruno Gomes, Fabiane G. Caldeira Brant, Camila J. Pereira-Pinto, Juliana P. Welbert, Jean P. S. Costa, Alexandra V. Yingling, Ivy Hurwitz, Mariana R. David, Fernando A. Genta

**Affiliations:** 1 Laboratório de Bioquímica e Fisiologia de Insetos, Instituto Oswaldo Cruz (IOC-Fiocruz), Rio de Janeiro, Brazil; 2 Instituto Nacional de Endemias Rurais (INERU-Fiocruz), Rio de Janeiro, Brazil; 3 Center for Global Health, University of New Mexico Health Sciences, Albuquerque, NM, United States of America; 4 Laboratório de Mosquitos Transmissores de Hematozoários, Instituto Oswaldo Cruz (IOC-Fiocruz), Rio de Janeiro, Brazil; 5 Instituto Nacional de Ciência E Tecnologia Em Entomologia Molecular, Rio de Janeiro, Brazil; Beni Suef University Faculty of Veterinary Medicine, EGYPT

## Abstract

The yeast-encapsulated orange oil (YEOO) is a novel larvicide under development against vector mosquitoes. Despite its efficiency against *Aedes aegypti* (L.) in small scale experiments, its applicability in vector control can be influenced by other effects on mosquito behaviour or physiology. For this reason, the impact of YEOO particles in mosquito oviposition was evaluated in laboratory and semi-field conditions. Oviposition assays with one gravid *Aedes aegypti* female were carried under laboratory and semi-field conditions with natural light and temperature fluctuation. For all ovitraps, the number of eggs was manually counted in the wooden paddle and in the solution of each ovitrap. The proportion of eggs between substrates (wooden paddle and solution) varied between conditions, with females in laboratory presenting a lower preference to lay eggs in paddles when compared with studies in semi-field. This behaviour shifts in laboratory can create challenges to extrapolate results from laboratory to the field. Here, studies in both conditions indicate a similar impact of YEOO particles in *Aedes aegypti* oviposition. The potential treatment concentration of YEOO particles presents a strong repellent/deterrent effect (-0.559 > OAI > -0.760) within the initial 72h of application when compared with water, and weak repellent/deterrent signal (OAI = -0.220) when compared against inactivated yeast. Control ovitraps with water were more positive for egg presence than treated ovitraps, while ovitraps with YEOO particles and inactivated yeast present similar number of positive ovitraps. It is possible that the repellent/deterrent action is partially driven by the delivery system, since most times *Citrus sinensis* EO oviposition repellent/deterrent signal is weak, and it seem influenced by solvent/delivery used. However, it is unclear how the yeast wall that protect/surrounds the orange oil will negatively affect oviposition since live yeast are normally consider an attractant for mosquito oviposition.

## Introduction

In 1901, General William C. Gorgas carried the first successful vector control campaign against *Aedes aegypti* (L.) in Havana (Cuba) by utilizing two key strategies: mosquito breeding site reduction and the implementation of mosquito nets to disrupt their biting behavior [1]. This accomplishment prompted similar campaigns across the Americas to eliminate yellow fever-causing mosquitoes (*Ae*. *aegypti*) from urban areas. In the 1940s, neurotoxic chemical insecticides, including organophosphates, played a pivotal role in *Ae*. *aegypti* eradication in many vector control campaigns [1]. However, the excessive use of these synthetic compounds, such as pyrethroids, has led to the development of resistance in natural mosquito populations worldwide, including in Brazil [2]. In recent years, there has been a shift towards more comprehensive approaches to tackling mosquito-borne disease. Integrated Vector Management (IVM) strategies is a coordinated approach that utilizes various interventions that target mosquitoes throughout their life cycle to minimize their impact on human health [3]. To further enhance IVM strategies, innovative tools are actively being developed for use as larvicides and adulticides with the goal of reducing the reliance on neurotoxic insecticides [4].

Essential oils (EO) are hydrophobic liquids extracted from plants that are rich in secondary/specialized metabolites generally associated with a defensive role against pathogens or herbivores. Some of these oils have been extensively studied for their insecticidal efficiency against mosquitos and for alternative applications in vector control. Citronella oil (*Cymbopogon nardus*), for example, is a well-known mosquito repellent, and is readily available commercially [5, 6]. EO from more than 27 plant families, especially Lamiaceae, Cupressaceae, Rutaceae, Apiaceae, and Myrtaceae, also exhibit larvicidal efficacy against many mosquito species [7, 8]. However, their practical application as such in the field is challenging. Their hydrophobic nature makes them unsuitable for direct application into aquatic environments without causing disruptions to the ecosystem. Further, EOs are susceptible to rapid degradation by ultraviolet (UV) radiation, temperature, and oxidation. Our team has tackled this challenge by encapsulating selected EOs into *Saccharomyces cerevisiae* (bakers’ yeast), a commonly used biocompatible and biodegradable container for a variety of exogenous compounds [9]. In previous studies, yeast-encapsulated orange oil (YEOO) was shown as a novel larvicide, with high efficiency (LC_50_<50 mg/L) against *Ae*. *aegypti* in small-scale laboratory trials [9, 10].

Although YEOO shows promise as a potential mosquito control agent, its suitability in IVM may be influenced by its impact on other mosquito behaviours. Among these behaviours is oviposition, where female mosquitoes actively seek suitable breeding sites to deposit their eggs. Several EO from different plant families as Poaceae, Myrtaceae, Rutaceae, Apiaceae, Piperaceae, Lamiaceae, Lauraceae, and Verbenaceae, have been shown as effective oviposition deterrents [11–14]. If YEOO can also disrupt this critical behaviour, it has the potential to prevent egg deposition in treated areas, leading to a reduction in the mosquito population. Additionally, by discouraging female mosquitoes from selecting treated sites for egg-laying, YEOO can help decrease mosquito density in specific locations. This multi-faceted impact on mosquito behaviours makes YEOO a promising candidate for comprehensive and location-specific mosquito control strategies within the context of IVM. In the following study, the impact of YEOO in the oviposition behaviour of *Ae*. *aegypti* females under laboratory and semi-field conditions was shown.

## Materials and methods

### Mosquito and product descriptions

*Ae*. *aegypti* (Rockefeller) eggs were provided by a reference laboratory for insecticide susceptibility tests at the Oswaldo Cruz Institute (Laboratório de Biologia, Controle e Vigilância de Insetos Vetores; IOC-Fiocruz). Eggs were hatched in filtered tap water with a yeast pallet (500 mg). Larvae were maintained under controlled laboratory conditions (i.e., 28 ± 2°C, 12/12 regulated light with white fluorescent lamps), and were fed approximately 250 mg/day of fish food (TetraMin, Tetra, Spectrum Brands Company, WI, USA) per 2,000 larvae daily. Adults were maintained in cages at controlled laboratory conditions with a 10% sucrose solution. Blood-feeding was offered to mosquitoes after 48 h of emergence. Human blood was provided to females by a cotton path on a flask with warm water for approximately 30–45 min. All engorged females were separated and maintained with 10% sucrose for 3–4 days until the beginning of oviposition bioassays. Mosquito rearing and blood feeding was performed under license L-4/2008 of the Animal Use and Ethics Committee of the Oswaldo Cruz Institute (CEUA-IOC/Fiocruz).

Yeast encapsulated orange oil (YEOO) was synthesized by encapsulation of *Citrus sinensis* EO (CAS Number: 8008-57-9; Cold pressed from peel fruit; Limonene as main component) inside *S*. *cerevisiae* (Red Star fresh baker’s yeast) as described by Workman *et al*. [9]. Lyophilized YEOO was rehydrated to 50 mg/L of orange oil in water. For the bioassays, the working concentration was 160 mg/L, equivalent to 10x the LC_90_ determined for third instar (L3) *Ae*. *aegypti* larvae [9]. Inactivated yeast (IY) was prepared by mixing 1 g *S*. *cerevisiae* in 4.5 ml water. The yeast was inactivated by heating (≈70°C) and mixing the solution for 20-30min without boiling [9].

### Oviposition bioassays

Two different experimental conditions were used to determine the impact of YEOO on *Ae*. *aegypti* oviposition: 1) Laboratory conditions: “YEOO *vs*. water” inside BugDorm-2400 Insect Rearing Tent (W75 x D75 x H115 cm); and 2) Semi-field conditions with natural light and temperature fluctuation: “YEOO *vs*. water”, “YEOO *vs*. IY” or “IY *vs*. water” inside Pop-up Cage with sleeves (W60 x D60 x H90 cm, Product ID: E60981, Walking & Doncaster, UK).

For the laboratory-based trials, assays were performed in two testing rooms with an ambient temperature of 28 ± 3°C. A 12h light/dark regime with white fluorescent lamps was employed in room 1. In room 2, the photoperiod was unavailable, and the oviposition assay was performed in the dark except during mosquito manipulation (*i*.*e*., setup and removal of ovitraps). Four ovitraps (black pot + wooden paddle) were loaded with 250 ml solution. A suspension of YEOO (160 mg/L) was added to two ovitraps. As control, an equivalent amount of water was added to the remaining traps. The ovitraps were then placed at each corner of the BugDorm-2400. The placement pattern (control vs. treatment) has a diagonal pattern that varies across each assay to avoid bias associated with ovitrap location. For each trial, a single gravid female (3–4 days after bloodmeal) was released into each BugDorm. After 48 h or 72h, the wooden paddle was collected from each ovitrap. The solution within the ovitrap was collected and the inside of the ovitrap was then rinsed with water to recover any eggs that may have been deposited on the plastic walls of the ovitrap. Eggs on the paddle and those collected from the solution from the ovitrap including those from the rinses were manually counted. These experiments were repeated five times using six BugDorms, with varying ovitrap positions on each run. All 72h assays were carried in “room 1” with the 12:12 photoperiod, while all 48h assays were carried in “room 2” without light pattern (darkness).

For the semi-field trials, oviposition assays were performed inside 16 Pop-up Cages with sleeves (W60 x D60 x H90 cm) with natural light and temperature fluctuation in two locations. Location 1 was a courtyard protected from rain with natural light and air circulation, while location 2 was a room with a large window with natural light and restricted air circulation. Location 2 was used only for one trial due to termite infestation that required insecticide intervention. In these assays, each ovitrap (black pot + wooden paddle) was loaded with 250 ml solution. Traps each were loaded with either a suspension of YEOO (160 mg/L), a suspension of IY (equivalent number of particles to YEOO at 160 mg/L, approx. 870 μL from stock solution), or an equivalent amount of water (control). Three combinations were used to determine the effect of YEOO on oviposition—YEOO *vs*. water; YEOO *vs*. IY and IY *vs*. water. As previously described, two ovitraps of each treatment were placed in a diamond pattern at the corner of a Pop-up Cage. Ovitrap placement pattern varied between assays to avoid bias associated with ovitrap location. One gravid female (3–4 days after blood meal) was placed inside each tent to infer the number of eggs laid during 72 h for each trial. Eggs were collected as described above. Further, temperature, humidity, and light intensity were recorded every 10 min throughout each oviposition experiment (S1–S3 Figs) using a HOBO U12 Temp-RH-Light-External Logger (Onset Computer Corp, MA, USA). This experiment was repeated four times with varied ovitrap placement at each trial.

The assays presented a high success rate with most assays presenting eggs in at least one ovitrap. In the laboratory-based trials, eggs were present in all BugDorms except for two in the 72h assays (15/15 for 48h; and 13/15 for 72h). A similar pattern was observed in assays performed under semi-field conditions. Eggs were present in 85.7% of the YEOO *vs*. water assays (12/14), 95.2% of the YEOO *vs*. IY assays (20/21), and 80.9% of IY *vs*. water assays (17/21). The highest number of assays where no eggs were found (*N* = 4) were in cages containing inactivated yeast and water.

### Data analysis

The presence of eggs in any of the ovitraps, treated or control, is counted as a valid assay, while the absence of eggs renders the assay invalid, and it is removed from the analysis. Fisherʼs exact tests were performed using the online platform Scistat (https://www.scistat.com/statisticaltests/fisher.php) to determine whether control or treated ovitrap placement affected oviposition. The difference in the number of eggs among substrates (paddle or solution) per treatment was determined using the Wilcoxon matched-pairs single rank test in Prism 9 (GraphPad Software, CA, USA).

Chi-square tests were used to identify differences in the egg-laying patterns under the various treatment conditions, i.e.: water/IY/YEOO alone, or mixed treatments. The oviposition activity index (OAI) was also calculated [15] defined by Eq 1 to infer the attractivity/stimulation *vs*. repellence/deterrence of the substance to oviposition. The index uses the number of eggs in experimental treatment (*Nt*), and the number of eggs in control (*Nc*) and varies between -1 and 1. The positive values suggest an attractive/stimulant effect for oviposition, while negative values indicate a potential repellent/deterrent activity. The oviposition index (OAI) was calculated between substrates and for YEOO *vs*. water, YEOO *vs*. IY, and IY *vs*. water.

**Eq 1.** Oviposition activity index—OAI [15]

$$OAI=\frac{Nt-Nc}{Nt+Nc}$$

Generalized Linear Models (GLMs) with binomial distribution were fitted separately for the two experimental settings (laboratory or semi-field conditions) to determine factors that predict egg-laying behaviour. For the laboratory setting, the assay duration (48 or 72h), ovitrap position within the cage, and treatment (YEOO *vs*. water) were included as independent variables. For semi-field assays, ovitrap position within the cage, the cage location (courtyard or room), and treatment (YEOO *vs*. water; YEOO *vs*. IY; or IY *vs*. water) were included as independent variables. The assumptions of the best model were examined by checking heteroscedasticity, residual dispersion, and the presence of outliers using the R package DHARMa [16].

To compare the number of eggs laid per treatment, GLMs with negative binomial distribution were fitted for the two experimental settings (laboratory and semi-field conditions). The total number of eggs from each ovitrap (per substrate and total number) was included as dependent variable. For the laboratory setting, the assay duration (48 or 72h), ovitrap position within the cage, and the treatment condition (YEOO *vs*. water) were included as independent variables. For the semi-field environment, ovitrap position within the cage, the cage location (courtyard or room) and treatment (YEOO *vs*. water; YEOO *vs*. IY; IY *vs*. water) were included as independent variables. Regressions with negative binomial distribution were preferred over the traditional Poisson distribution because data exhibited over-dispersion (i.e., variance was larger than the mean), confirmed by the DHARMa nonparametric dispersion test, performed using the ‘DHARMa’ R package [16]. The consistency of data with the negative binomial distribution was verified using the goodness of fit test ‘Minimum Chi-squared’ (*p*-value > 0.05) from the ‘goodfit’ command, implemented in the ‘vcd’ R package [17]. The assumptions of the best model were examined by checking heteroscedasticity, residuals dispersion, and the presence of outliers using the R package DHARMa [16].

## Results

### Substrate preference

**Fig 1:**
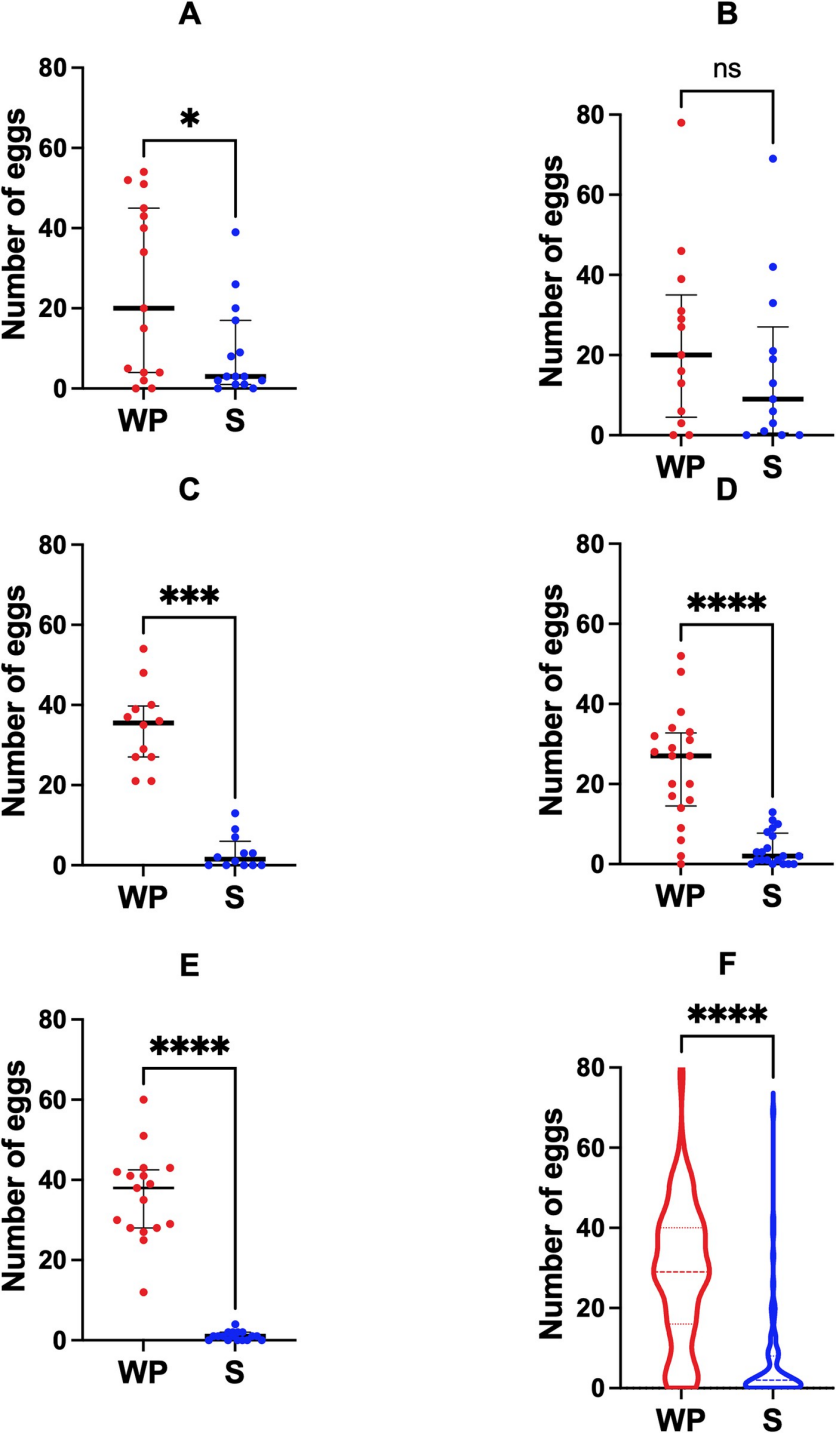
Distribution of number of mosquito eggs by substrate in five oviposition experiments. (A) 48h assays YEOO *vs*. water under laboratory conditions, (B) 72h assays YEOO *vs*. water under laboratory conditions, (C) 72h assays YEOO *vs*. water at semi-field conditions with natural light, (D) 72h assays YEOO *vs*. IY at semi-field conditions with natural light, (E) 72h assays IY vs. water at semi-field conditions with natural light, (F) violin plot of all data. Black lines: median with interquartile range; red dots (WP): eggs counted in wooden paddle; blue dots (S): eggs counted in solution of the ovitrap; ns: *p* > 0.05; *: *p* < 0.05; **: *p* < 0.01; ***: *p* < 0.001; ****: *p* < 0.0001.

The number of eggs on the wooden paddles and in solution were determined separately for each ovitrap. In 66.2% of the assays, eggs were found in both in solution and on the paddle. In five assays (6.5%), there were no eggs on the wooden paddles. Conversely, in 21 assays, there were no eggs in solution (27.3%). As expected, most assays present more eggs laid on the wooden paddles compared to those observed in the ovitrap solution and plastic surfaces of the ovitrap (alternative surface) in both the laboratory-based and semi-field trials (Fig 1A–1F, Wilcoxon matched-pairs signed-rank test: W = -2,440; *P* < 0.0001). Overall, a positive oviposition activity index was computed in favour of paddles (OAI = 0.639, Table 1). The “YEOO *vs*. water” 72h laboratory assays were the exception to this pattern presenting a lack of variation between paddle and alternative surface (Fig 1B, Wilcoxon matched-pairs signed-rank test: W = -29; *P* = 0.330). The proportion of eggs found between the wooden paddles and alternative surface was different between the laboratory and semi-field trials (*χ*^*2*^ *=* 287.52, *df =* 2, *P* = 0.0076). The laboratory-based OAI was 2.6-fold smaller than that of the assays performed under semi-field conditions (OAI-LAB = 0.318 *vs*. OAI-SF = 0.839). From here, all remaining calculations show results that combine egg counting from both substrates, while calculations based on substrates are in the S1 Text.

**Table 1:** Oviposition activity index for different substrates in laboratory and semi-field conditions. In each ovitrap tested, eggs laid in the paddle, and eggs laid in the solution or in the plastic surfaces were manually counted, separately. In each condition, the Oviposition Activity Index (OAI) was calculated considering the paddle as the experimental group and the other conditions (named as alternative surface) as the control. A separate analysis was performed grouping the data from laboratory and semi-field conditions (named “overall”). *Nt*: the sum number of eggs in experimental treatment; *Nc*: the sum of number of eggs in control; alternative surface: solution and plastic surfaces of the ovitrap; OAI: Oviposition activity index; Laboratory: assays under laboratory conditions for both 48h and 72h; Semi-field: 72h assays at environmental conditions with natural light.

Comparison	Conditions	Experiment treatment	*Nt*	Control	*Nc*	OAI
paddle vs. solution	Laboratory	paddle	677	alternative surface	350	0.318
paddle vs. solution	Semi-field	paddle	1509	alternative surface	132	0.839
paddle vs. solution	Overall	paddle	2186	alternative surface	482	0.639

### Presence vs. absence of eggs in ovitraps

**Fig 2:**
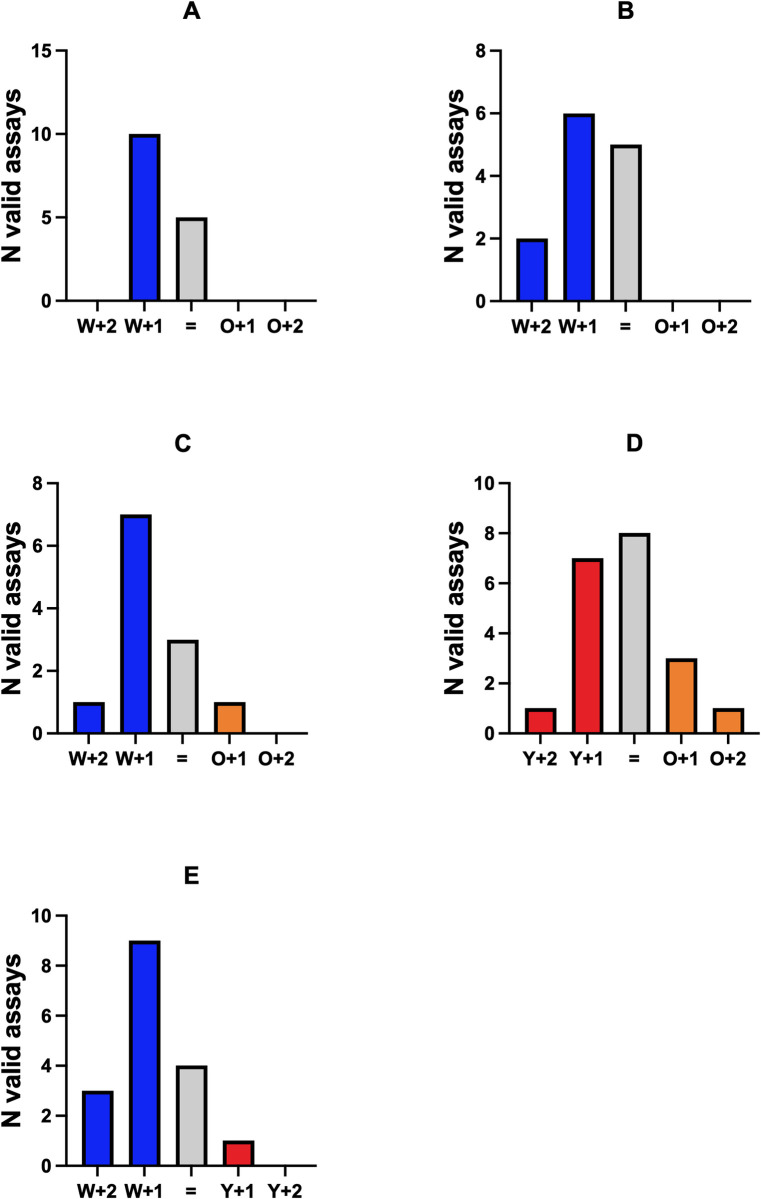
Distribution of positive ovitraps in valid assays. (A) 48h assays YEOO *vs*. water under laboratory conditions, (B) 72h assays YEOO *vs*. water under laboratory conditions, (C) 72h assays YEOO *vs*. water at semi-field conditions with natural light, (D) 72h assays YEOO *vs*. IY at semi-field conditions with natural light, (E) 72h assays IY vs. water at semi-field conditions with natural light. Blue bar: assays with more positive ovitraps with water (W+2: eggs two ovitraps with water; W+1: eggs in one ovitrap with water or eggs in three ovitraps with more positive ovitraps with water); Orange bar: assays with more positive ovitraps with YEOO (O+2: eggs in two ovitraps with YEOO; O+1: eggs in one ovitrap with YEOO or eggs in three ovitraps with more positive ovitraps with YEOO); Red bar: eggs found only in ovitraps with IY (Y+2: eggs in two ovitraps with IY; Y+1: eggs in one ovitrap with IY or eggs in three ovitraps with more positive ovitraps with IY); Light grey (=): eggs found in two or four ovitraps with mixed treatments.

The variation between number of positive ovitraps per assay did not significant varied among the five experiments (χ2 = 12.35, df = 12, *p* = 0.4183, Table 2). In both laboratory and semi-field conditions, ovitraps with water were more likely to contain eggs when compared to ovitraps treated with YEOO or IY (Fig 2). The impact of ovitrap treatment on oviposition was investigated by GLMs. Our initial regression model demonstrates that assay duration (48 or 72h) did not impact the presence of eggs in the traps (β-coefficient = 0.69, *P* = 0.11, Table 3). As such, all assays in semi-field were performed for 72h. Regression models for laboratory-based (β-coefficient = -1.55, *P* < 0.001) and semi-field (β-coefficient = -1.47, *P* = 0.04) assays with YEOO vs water suggest a preference for oviposition in traps filled with water instead of YEOO. Similar preference for water was identified between IY vs water (β-coefficient = -1.84, *P* = 0.001). Interestingly, female mosquitoes exhibited no preference for ovitraps filled with either YEOO or IY (β-coefficient = -0.38, *P* = 0.41) under semi-field conditions (Tables 2 and 3, Fig 2). In semi-field, location in YEOO vs water (β-coefficient = 2.3, *P* = 0.04) may be a contributing factor to the analyses. Thus, considering there were no consistent effects across all comparisons under semi-field conditions (no signal for IY *vs*. water: β-coefficient = -1.15, *P* = 0.14; or YEOO *vs*. IY: β-coefficient = -0.37, *P* = 0.56), the data from the “room” in our analyses was maintained. Separate GLM models developed for the number of eggs on the paddles and eggs in solution and other surfaces showed similar results (S1 Text).

**Table 2:** Description of the positive ovitraps based on the number of positive ovitraps per assay and type of treatment. Values in parenthesis refer to frequencies within the total number of valid assays in each comparison. YEOO: yeast-encapsulated orange oil; IY: Inactivated yeast; Lab (48h+72h): assays under laboratory conditions for both 48h and 72h; SF72h: 72h assays at semi-field conditions with natural light; One: number of assays with only one positive ovitrap inside the tent; Two: number of assays with two positive ovitraps inside the tent; Three: number of assays with three positive ovitraps inside the tent; Four: number of assays with four positive ovitraps inside the tent; W: number of assays only positive for ovitraps with water; O: number of assays only positive for ovitraps treated with YEOO; Y: number of assays only positive for ovitraps with IY; Mix: number of assays positive for ovitraps with different treatments; NA: not applicable; *: assays with two positive ovitraps agregate assays with a mix of positive ovitraps (one of each) or assays with 2 ovitraps of a specific treatment.

Comparison	Conditions	Number positive ovitraps				Positive ovitraps treatment			
		One	Two*	Three	Four	W	O	Y	Mix
YEOO vs. water	Lab48h	6 (0.400)	3 (0.200)	4 (0.267)	2 (0.133)	6 (0.400)	0 (0.0)	*NA*	9 (0.600)
YEOO vs. water	Lab72h	2 (0.153)	3 (0.231)	4 (0.308)	4 (0.308)	4 (0.308)	0 (0.0)	*NA*	9 (0.692)
YEOO vs. water	SF72h	7 (0.584)	3 (0.250)	1 (0.083)	1 (0.083)	7 (0.583)	1 (0.084)	*NA*	4 (0.333)
YEOO vs. IY	SF72h	5 (0.250)	7 (0.350)	5 (0.250)	3 (0.150)	*NA*	3 (0.150)	4 (0.200)	13 (0.650)
IY vs. water	SF72h	9 (0.529)	5 (0.294)	1 (0.059)	2 (0.118)	11 (0.647)	*NA*	1 (0.059)	5 (0.294)

**Table 3:** Results of the generalized linear models (binomial) of the presence of eggs in ovitraps. YEOO: yeast-encapsulated orange oil; IY: Inactivated yeast; Coefficient (ß): coefficient that represent the estimated change in the “dependent variable” impacted by the “independent variable”; the “independent variable” presents a significative impact when *p*-value < 0.05.

Dependent variable	Independent variable	Coefficient (ß)	Std. error	z-value	p-value
“YEOO vs. water” in the laboratory	(Intercept)	0.55	0.49	1.12	0.26
	Time (72H)	0.69	0.43	1.61	0.11
	Ovitrap position (2)	0.14	0.59	0.24	0.81
	Ovitrap position (3)	0.80	0.62	1.29	0.20
	Ovitrap position (4)	0.59	0.60	0.98	0.33
	**Treatment (YEOO)**	**-1.55**	**0.44**	**-3.54**	**< 0.001**
“YEOO vs. water” in the semi-field setting	(Intercept)	0.62	0.71	0.88	0.38
	Ovitrap position (2)	-1.91	1.09	-1.74	0.08
	Ovitrap position (3)	-0.43	0.93	-0.46	0.64
	Ovitrap position (4)	-0.42	0.94	-0.44	0.66
	**Treatment (YEOO)**	**-1.47**	**0.73**	**-2.01**	**0.04**
	**Location (Room)**	**2.30**	**1.10**	**2.10**	**0.04**
“YEOO vs. IY” in the semi-field setting	(Intercept)	1.09	0.55	1.97	0.05
	Ovitrap position (2)	-0.62	0.67	-0.92	0.36
	Ovitrap position (3)	-0.66	0.67	-0.98	0.33
	Ovitrap position (4)	-0.82	0.67	-1.23	0.22
	Treatment (YEOO)	-0.38	0.46	-0.83	0.41
	Location (Room)	-0.37	0.64	-0.58	0.56
“IY vs. water” in the semi-field setting	(Intercept)	0.86	0.61	1.40	0.16
	Ovitrap position (2)	-0.36	0.79	-0.46	0.65
	Ovitrap position (3)	0.32	0.80	0.40	0.69
	Ovitrap position (4)	-0.07	0.78	-0.09	0.93
	**Treatment (IY)**	**-1.84**	**0.56**	**-3.28**	**0.001**
	Location (Room)	-1.15	0.77	-1.48	0.14

There were only two valid assays without eggs on ovitraps with water: one from semi-field YEOO *vs*. water and one for IY *vs*. water. In the laboratory, assays with eggs in mixed treatment (60% for 48h assays, 69% for 72h assays) were more frequent than assays with eggs only in ovitraps filled with water (Table 2). On the other hand, under semi-field conditions, assays with positive ovitraps for mixed treatments were 33.3% for YEOO *vs*. water and 29.4% for IY vs. water (Table 2).

There were significantly more water-filled traps positive for eggs for 48h laboratory assays YEOO *vs*. water (*Fisherʼs exact test*: *P* = 0.0169), 72h semi-field assays YEOO *vs*. water (*Fisherʼs exact test*: *P* = 0.0143), and IY *vs*. water (*Fisherʼs exact test*: *P* = 0.0008). On the other hand, the number of eggs in the 72h laboratory assays “YEOO *vs*. water” were similar (*Fisherʼs exact test*: *P* = 0.0956). This is likely due to the higher proportion of assays with eggs in mixed treatments. In the YEOO *vs*. IY setting, positive ovitraps were observed for mixed treatments in 65.0% of assays (*Fisherʼs exact test*: *P* = 1.0). Three assays had only positive ovitraps with YEOO while four assays had only positive ovitraps with inactivated yeast.

In Table 2, the number of valid assays with eggs in ovitraps with mixed treatments and assays with eggs in only one of the treatments was counted. When the pattern of YEOO *vs*. IY was compared against assays with water as control, significant differences were found only between patterns for the comparison “YEOO *vs*. IY” *vs*. “IY *vs*. water” (χ2 = 9.748, df = 2, *p* = 0.0076). At the same time, the other three comparisons were not significant (χ2, df = 2, *p* > 0.05).

### Number of eggs

**Fig 3:**
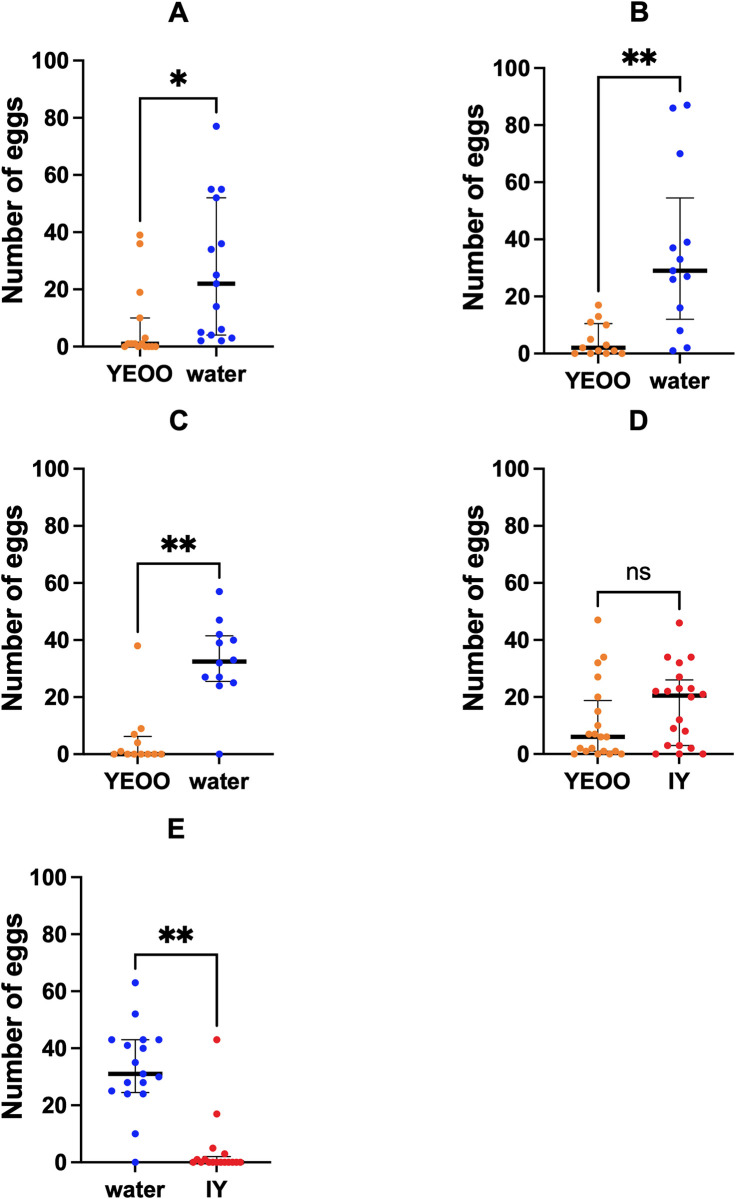
Distribution of number of mosquito eggs in five oviposition experiments. (A) 48h assays YEOO *vs*. water under laboratory conditions, (B) 72h assays YEOO *vs*. water under laboratory conditions, (C) 72h assays YEOO *vs*. water at semi-field conditions with natural light, (D) 72h assays YEOO *vs*. IY at semi-field conditions with natural light, E: 72h assays IY vs. water at semi-field conditions with natural light. Black lines: median with interquartile range; orange dots (YEOO): yeast-encapsulated orange oil; blue dots: water; red dots (IY): inactivated yeast; ns: *p* > 0.05; *: *p* < 0.05; **: *p* < 0.01; ***: *p* < 0.001; ****: *p* < 0.0001.

The total number (paddle as well as those observed in the water and rinses) of eggs from each ovitrap was utilized in the following analyses. The total number of eggs per ovitrap were significantly higher in ovitraps filled with water than in ovitraps with YEOO or inactivated yeast. This pattern was consistent for assays performed in laboratory and semi-field conditions (Fig 3). GLMs further confirmed the impact of the ovitrap treatment on the number of eggs, indicating the preference of females for laying more eggs in ovitraps with water instead of YEOO or inactivated yeast (Table 4).

**Table 4:** Results of the generalized linear models (negative binomial) of egg number in ovitraps. YEOO: yeast-encapsulated orange oil; IY: Inactivated yeast; Coefficient (ß): coefficient that represent the estimated change in the “dependent variable” impacted by the “independent variable”; the “independent variable” presents a significative impact when p-value < 0.05.

Dependent variable	Independent variable	Coefficient (ß)	Std. error	z-value	p-value
“YEOO vs. water” in the laboratory	(Intercept)	2.69	0.43	6.22	< 0.001
	Time (72H)	-0.13	0.36	-0.36	0.72
	Ovitrap position (2)	-0.00	0.52	0.01	0.99
	Ovitrap position (3)	0.30	0.51	0.58	0.56
	Ovitrap position (4)	0.02	0.52	0.04	0.97
	**Treatment (YEOO)**	**-1.57**	**0.37**	**-4.25**	**< 0.001**
“YEOO vs. water” in the environment setting	(Intercept)	3.23	0.69	4.65	< 0.001
	**Ovitrap position (2)**	**-3.54**	**1.08**	**-3.28**	**0.001**
	Ovitrap position (3)	-1.08	0.91	-1.18	0.24
	Ovitrap position (4)	-0.93	0.94	-0.99	0.32
	**Treatment (YEOO)**	**-1.47**	**0.72**	**-2.04**	**0.04**
	**Location (Room)**	**2.26**	**0.89**	**2.55**	**0.01**
“YEOO vs. IY” in the environment setting	(Intercept)	2.20	0.47	4.73	< 0.001
	Ovitrap position (2)	-0.51	0.60	-0.86	0.39
	Ovitrap position (3)	0.11	0.59	0.19	0.85
	Ovitrap position (4)	0.11	0.59	0.18	0.86
	Composition (YEOO)	-0.47	0.42	-1.11	0.27
	Location (Room)	-0.06	0.59	-0.10	0.92
“IY vs. water” in the environment setting	(Intercept)	2.87	0.63	4.56	0.00
	Ovitrap position (2)	0.31	0.80	0.39	0.70
	Ovitrap position (3)	1.16	0.80	1.46	0.14
	Ovitrap position (4)	-0.21	0.81	-0.26	0.79
	**Treatment (IY)**	**-2.87**	**0.57**	**-5.06**	**< 0.001**
	Location (Room)	-0.65	0.75	-0.87	0.39

For all pairwise comparisons, the number of eggs were higher in water-filled ovitraps when compared to YEOO (Lab: β-coefficient = -1.55, *P* < 0.001; and semi-field: β-coefficient = -1.47, *P* = 0.04) and IY (β-coefficient = -2.87, *P* < 0.001). The median number of eggs laid in water-filled ovitraps per assay vary between 22.0 (IQR = 4.0–52.0) and 32.5 (IQR = 25.5–41.5), contrasting with lower range in YEOO from 0.0 (IQR = 0.0–6.3) to 2.0 (IQR = 0.0–10.5) and lower value in inactivated yeast (0.0, IQR = 0.0–2.0). For assays where YEOO was tested against IY, the number of eggs in ovitraps with IY (median = 20.5, IQR = 3.0–26.0) was higher than in ovitraps treated with YEOO (median = 6.0, IQR = 1.0–18.8). However, this variation was not significant (β-coefficient = -0.38, *P* = 0.41, Fig 3, Table 4, and S1 Table).

The GLMs did not suggest any consistent influence of other variables on the total number of eggs. The semi-field “YEOO *vs*. water” model suggests that location (β-coefficient = 2.26, *P* = 0.01) and ovitrap position 2 (β-coefficient = -3.54, *P* = 0.001) may influence the number of eggs laid. However, the median number of eggs per assay in the “YEOO *vs*. water” comparison under semi-field conditions were similar between both locations (“room”: 37.0 per assay; “courtyard”: 37.5 per assay). It is possible that the discrepancies between the number of assays in the “room” (*N* = 2) versus “courtyard” (*N* = 10) might have created a statistical artifact in the model. Similarly, the effect of ovitrap position in the model from YEOO *vs*. water in the semi-field may be due to the removal of invalid assays (Table 4). Under this condition, eight assays were performed with YEOO in position 2/4 and eight in position 1/3. All invalid assays had YEOO in position 1/3, causing a higher number of valid assays with YEOO in position 2 and 4 (8 out of 12) with only four assays with water in these positions. A higher proportion of assays with YEOO increased the number of assays without eggs or low counting in position 2 and 4, while all four assays with water are only positive for one ovitrap (3 positive for position 4, and 1 positive for position 1), which led to lower number of eggs in position 2 than other positions. Further, this effect was insignificant in the other combinations of treatments. For this reason, this was likely a residual effect without any biological value. Fewer YEOO *vs*. water assays performed under semi-field conditions were performed. As such, the interpretation of this model requires caution. Moreover, “location” in the comparison YEOO *vs*. water was not significant for wooden paddles, the substrate with the higher overall number of eggs (S1 Text).

Overall, the oviposition activity index (OAI) of YEOO was negative for all four comparisons with water (from -0.760 to -0.559) and inactivated yeast (-0.220), suggesting a repellent/deterrent effect of this larvicide for *Aedes aegypti* oviposition. However, a negative OAI (-0.778) was also observed for IY *vs*. water, suggesting a similar repellent/deterrent effect. The OAI of YEOO *vs*. IY was still negative (-0.220) but closer to the reference zero, when compared with the OAI from YEOO *vs*. water (Table 5). This scenario suggests a lower repellent/deterrent effect on oviposition for YEOO than comparisons with water.

**Table 5:** Oviposition activity index for each comparison between different ovitrap compositions. For each comparison, one condition is defined as Experimental and the other as Control. Total eggs in each ovitrap were manually counted, including the eggs in paddles, solutions and plastic surfaces. The results of all experimental replicates were then grouped. *Nt*: the sum number of eggs in the experimental treatment; *Nc*: the sum of number of eggs in control; OAI: Oviposition activity index; YEOO: yeast-encapsulated orange oil; IY: inactivated yeast; Lab48h, and Lab72h: assays under laboratory conditions for 48h or 72h, respectively; SF72h: 72h assays at semi-field conditions with natural light.

Comparison	Conditions	Experimental treatment	*Nt*	Control	*Nc*	OAI
YEOO vs. water	Lab48h	YEOO	111	water	392	-0.559
YEOO vs. water	Lab72h	YEOO	63	water	461	-0.760
YEOO vs. water	SF72h	YEOO	59	water	393	-0.739
YEOO vs. IY	SF72h	YEOO	218	IY	341	-0.220
IY vs. water	SF72h	IY	70	water	560	-0.778

## Discussion

Yeast-encapsulated orange oil (YEOO) demonstrated a repellent/deterrent effect for the ***oviposition*** of *Ae*. *aegypti* (Rockefeller lineage), which was consistent in all comparisons against water (OAI: strongly negative [- 0.76, - 0.56]) and with a lower number of eggs in positive YEOO-ovitraps. YEOO is a novel larvicide with high efficiency against *Ae*. *aegypti* [9, 10], and it is vital to understand its alternative impacts on mosquito vectors, particularly in oviposition behaviour. Substances with attractive/stimulant properties for oviposition can potentially improve trapping systems for gravid females by improving egg-laying in the traps while preventing it in other water reservoirs. In contrast, repellent/deterrent for oviposition may be used to protect sites typically used by mosquitoes as larval breeders, especially those that cannot be removed from their environment or have economic value (e.g., drinking water supplies; industrial or commercial products such as tires and plants).

Oviposition is a fundamental phenomenon in the mosquito life cycle. The oviposition site selection by the gravid female involved multiple environmental factors including the availability of food, the presence of predators, and the proportion of competitors. Olfactory cues are typically considered the most important stimulus for oviposition since they guide female mosquitos toward the oviposition site during flight (attractive vs. repellent), and may facilitate the decision to lay eggs when they are on the substrate (stimulant vs. deterrent) [18]. Other stimuli also play a role in *Ae*. *aegypti* oviposition. Following landing in the water, female mosquitos will walk to seek moist substrates that are in contact with water to lay their eggs. Movement on the surface of the water allows the insect the opportunity to use tactile and gustatory receptors to decide on oviposition [19]. Substances or tools that affect oviposition are important for the development of control measures. Attractants, that encourage flight to the breeding site, or stimulants that promote egg laying, can be critical for the optimization of traps geared to lure gravid females. For *Aedes* mosquitoes, ovitraps have been a very efficient tool for surveillance programs to verify mosquito dynamics in regions with established populations (e.g., monitoring densities, supporting release control programs) and to identify new colonisations in high-risk areas (e.g., airports, shipping facilities). On the other hand, substances promoting repellence (which discourages flight to the breeding site) or deterrence (which inhibits laying eggs when landing on the substrate) have the potential to delay the recolonization of treated breeding sites. These characteristics can complement the action of insecticides in breeding sites that mechanical measurements cannot remove. Moreover, oviposition repellence/deterrence may also negatively impact control measures for the same motive, and their use should be evaluated cautiously. For example, the repellent/deterrent clues that avoid oviposition in some situations may undermine the action of other control measures when alternative breeding sites exist in proximity, leading to dispersion with mosquitoes laying eggs in untreated breeding sites allowing their normal development. Still, this limitation is shared with other control methods focusing on breeding sites, and it is always essential to combine different tools to achieve an ideal control outcome [20].

The repellent/deterrent signal observed in this study seems partially driven by the yeast delivery system. A similar repellent/deterrent signal was evident when comparing IY *vs*. water. In contrast, the slight variation observed between YEOO *vs*. IY was not significantly different in both the presence/absence data and the total number of eggs. This potential repellent/deterrent effect of the inactivated yeast is unexpected, given that yeast suspensions are commonly used as attractants for mosquito oviposition [21], and mosquito larvae commonly ingest yeast and their presence provides a higher food availability in the breeding site [22]. Most previous studies use live yeast, and it is possible that the inactivated yeast or the non-functional yeast wall (the only part of the yeast that survives the encapsulation process) presents a different dynamic in the water changing its effect in oviposition (e.g., lacks fermentation or production of metabolites). If the delivery system drives the repellent/deterrent pattern of YEOO, it will probably promote a consistent signal across different essential oil encapsulated in yeast. Further studies are necessary to clarify this topic.

The impact of orange oil as a repellent/deterrent remains inconclusive based on this study. When compared to IY, YEOO has weak repellent/deterrent effect (OAI = -0.22) and a slightly different distribution in the number of eggs but without statistical significance. For this reason, YEOO and inactivated yeast may have a similar influence on oviposition. The weak oviposition repellent/deterrent effect for *Citrus sinensis* EO has been demonstrated by other investigators. Specifically, Araujo et al. showed deterrent activity at 81.44 ppm (4x LC99 in the study) using Tween80 as the surfactant in Brazil (OAI = -0.2) [23], while Phasomkusolsil and Soonwera reported similar activities with *Citrus sinensis* EO with 5% soybean oil as the solvent in Thailand [24]. The repellent/deterrent effect of *Citrus sinensis* EO varies in the literature, the highest signal of *Citrus sinensis* EO (5–200 ppm) was observed when DMSO was used as the solvent in Colombia (OAI < -0.78) [25]. Contrasting results were seen in the study in Thailand [24], where OAI values are dependent on EO concentration. Here, lower EO concentrations (1%) had a weak attractive/stimulant signal (OAI = 0.1) while higher EO concentrations (10%) showed significant repellent/deterrent signals (OAI = -0.4). It should be noted that it is very challenging to compare the results of different studies as the composition of *Citrus* EOs will vary depending on the source/region [23, 25–27]. Nevertheless, the combination of our results and the literature suggests a weak oviposition repellence/deterrence activity for orange oil. However, the applicability of orange oil as a deterrent for oviposition may be dependent on the manner it is to be delivered to the target site. Furthermore, this deterrence may be correlated to specific molecules or plant metabolites that are present in *Citrus sinensis* oil. This can be accomplished by testing the deterrent properties of the primary components of *Citrus sinensis* oil such as limonene and myrcene.

In this study, only the effect of yeast-encapsulated essential oils on oviposition was investigated. There are currently no other methods to effectively deliver EO to mosquito breeding sites. The hydrophobic properties of the EO, when used directly, cause a physical barrier in the water, presenting a different killing mechanism from YEOO. The use of oils as physical barriers in mosquito breeding sites is well known and was widely used as a common vector control strategy before the emergence of chemical insecticides [1]. Nowadays, barriers with oils are normally avoided due to their detrimental environmental impact. However, some mineral oils and films are still authorized for this purpose. Further, EOs will rapidly degrade when exposed to natural light.

Ovitraps are essential for monitoring *Ae*. *aegypti* populations and can also be used in the surveillance of *Aedes*-borne viruses. As expected, most of the eggs laid by females were in a humid wooden substrate. Still, a high proportion of assays with eggs in the solution was observed (72.7%), indicating that mosquitoes laid eggs in an alternative location (directly in the solution or in the plastic pot of the ovitrap) with some frequency, or our handling of the ovitraps may cause some eggs to fall in the solution. This pattern was already observed for *Ae*. *aegypti* [19, 28]. In our laboratory assays, the variation of egg counting between wooden paddles and the solution was smaller than in assays under semi-field conditions, with 72h laboratory assays YEOO *vs*. water presenting no significant differences between substrates. This suggests that assays under laboratory conditions may show a behaviour shift compared to less controlled conditions. This tendency should be taken into consideration before the extrapolation of information about mosquito behaviour from oviposition data obtained in the laboratory. Most assays show a higher proportion of mosquitoes laying eggs in multiple ovitraps, which is consistent with the previous description of a tendency for skip-oviposition behaviour in *Ae*. *aegypti* [29, 30]. However, mosquitoes laying eggs in only one ovitrap were also frequent, with 29% to 40% of the females manifesting this behaviour.

YEOO at 160 mg/L presents a repellent/deterrent effect for *Ae*. *aegypti* oviposition within 48-72h of application. New experiments under field conditions should allow for verifying the extension of this effect in treated breeding sites and evaluating its duration during vector control activity. Overall, the oviposition activity index (OAI) of YEOO was negative for all comparisons with water (from -0.760 to -0.559) and inactivated yeast (-0.22), indicating a weak to strong repellent/deterrent effect for *Aedes* oviposition.

## Supporting information

S1 Text Results from generalized linear models based on data from different substrates: Wooden paddles and solution. (DOCX)

S1 Fig Temperature, humidity, and light intensity for location 1 (courtyard) between 17^th^ and 21^st^ January 2020. Location 1 is a courtyard protected from rain with natural light and air circulation. Black: temperature (°C), Blue: relative humidity (%), Green: light intensity (Lux).
(TIF)

S2 Fig Temperature, humidity, and light intensity for location 2 (room) between 17^th^ and 21^st^ January 2020. Location 2 is a room with a large window with natural light and restricted air circulation. Black: temperature (°C), Blue: relative humidity (%), Green: light intensity (Lux).
(TIF)

S3 Fig Temperature, humidity, and light intensity for location 1 (courtyard) between 10^th^ and 22^nd^ February 2020. Location 1 is a courtyard protected from rain with natural light and air circulation. Black: temperature (°C), Blue: relative humidity (%), Green: light intensity (Lux).
(TIF)

S1 Table Descriptive statistic of mosquito eggs in five oviposition experiments. Lab48h: 48h assays under laboratory conditions; Lab72h: 72h assays under laboratory conditions; Env72h: 72h assays at environmental conditions with natural light.
(DOCX)
