# Supplementary material for: The impact of yeast-encapsulated orange oil in Aedes aegypti oviposition
Source: PLoS One. 2024 May 14;19(5):e0301816. doi: 10.1371/journal.pone.0301816 (PMC11093346; doi:10.1371/journal.pone.0301816)
Supplement: S1 Text — (DOCX) [file pone.0301816.s001.docx]

**Text S1. Results from generalized linear models based on data from different substrates: wooden paddles and solution.**

- **Presence/absence data**

Experiments performed in both laboratory and semi-field presented lack of significant differences in the presence/absence of eggs in both substrates (i.e. wooden paddles and solution) regarding the assay duration or the ovitrap position inside the cage (Table A-B). For paddles, only treatment present a significant signal in all comparisons with water (more positive ovitraps with only water), while signals for solution present lower consistence. Location exhibited marginally significant differences for the comparison “YEOO *vs*. water” in semi-field conditions, and treatment was significative for “YEOO vs. water” in the laboratory and “YEOO vs. IY” (Table B). The only consistent signal between both substrates were only found in the laboratory, where oviposition attractivity towards YEOO was lower.

**Table A.** Results of the generalized linear models (binomial) of the presence of eggs on paddles in ovitraps.

| **Dependent variable** | **Independent variable** | **Coefficient (ß)** | **Std. error** | **z-value** | **p-value** |
| --- | --- | --- | --- | --- | --- |
| “YEOO vs. water” in the laboratory | (Intercept) | -0.17 | 0.47 | -0.35 | 0.72 |
|  | Time (72H) | 0.04 | 0.40 | 0.09 | 0.93 |
|  | Ovitrap position (2) | 0.38 | 0.57 | 0.67 | 0.50 |
|  | Ovitrap position (3) | 0.40 | 0.57 | 0.70 | 0.48 |
|  | Ovitrap position (4) | 0.47 | 0.58 | 0.81 | 0.42 |
|  | **Treatment (YEOO)** | **-1.02** | **0.41** | **-2.51** | **0.01** |
| “YEOO vs. water” in the semi-field setting | (Intercept) | 0.85 | 0.71 | 1.20 | 0.23 |
|  | Ovitrap position (2) | -2.46 | 1.25 | -1.96 | 0.05 |
|  | Ovitrap position (3) | -0.42 | 0.92 | -0.45 | 0.65 |
|  | Ovitrap position (4) | -0.58 | 0.95 | -0.61 | 0.54 |
|  | **Treatment (YEOO)** | **-1.79** | **0.75** | **-2.38** | **0.02** |
|  | Location (Room) | 0.78 | 1.00 | 0.78 | 0.43 |
| “YEOO vs. IY” in the semi-field setting | (Intercept) | 1.07 | 0.55 | 1.94 | 0.05 |
|  | Ovitrap position (2) | -0.83 | 0.67 | -1.25 | 0.21 |
|  | Ovitrap position (3) | -0.86 | 0.67 | -1.29 | 0.20 |
|  | Ovitrap position (4) | -1.04 | 0.67 | -1.55 | 0.12 |
|  | Treatment (YEOO) | -0.27 | 0.46 | -0.58 | 0.56 |
|  | Location (Room) | -0.60 | 0.65 | -0.92 | 0.36 |
| “IY vs. water” in the semi-field setting | (Intercept) | 0.72 | 0.63 | 1.14 | 0.25 |
|  | Ovitrap position (2) | -0.37 | 0.86 | -0.43 | 0.67 |
|  | Ovitrap position (3) | 0.72 | 0.86 | 0.84 | 0.40 |
|  | Ovitrap position (4) | -0.02 | 0.85 | -0.03 | 0.98 |
|  | **Treatment (IY)** | **-2.44** | **0.63** | **-3.89** | **< 0.001** |
|  | Location (Room) | -0.92 | 0.81 | -1.13 | 0.26 |

YEOO: yeast-encapsulated orange oil; IY: Inactivated yeast

**Table B.** Results of the generalized linear models (binomial) of the presence of eggs on the solution inside ovitraps.

| **Dependent variable** | **Independent variable** | **Coefficient (ß)** | **Std. error** | **z-value** | **p-value** |
| --- | --- | --- | --- | --- | --- |
| “YEOO vs. water” in the laboratory | (Intercept) | -0.14 | 0.49 | -0.28 | 0.78 |
|  | Time (72H) | 0.65 | 0.43 | 1.52 | 0.13 |
|  | Ovitrap position (2) | -0.17 | 0.61 | -0.28 | 0.78 |
|  | Ovitrap position (3) | 0.93 | 0.60 | 1.56 | 0.12 |
|  | Ovitrap position (4) | -0.09 | 0.61 | -0.14 | 0.89 |
|  | **Treatment (YEOO)** | **-1.54** | **0.44** | **-3.52** | **< 0.001** |
| “YEOO vs. water” in the semi-field setting | (Intercept) | -0.83 | 0.72 | -1.25 | 0.25 |
|  | Ovitrap position (2) | -0.75 | 1.11 | -0.67 | 0.50 |
|  | Ovitrap position (3) | -2.05 | 1.35 | -1.52 | 0.13 |
|  | Ovitrap position (4) | -0.18 | 1.03 | -0.18 | 0.86 |
|  | Treatment (YEOO) | -0.80 | 0.88 | -0.91 | 0.36 |
|  | **Location (Room)** | **1.99** | **0.93** | **2.15** | **0.03** |
| “YEOO vs. IY” in the semi-field setting | (Intercept) | 0.13 | 0.55 | 0.24 | 0.81 |
|  | Ovitrap position (2) | -0.33 | 0.75 | -0.44 | 0.66 |
|  | Ovitrap position (3) | -0.00 | 0.72 | -0.00 | 1.00 |
|  | Ovitrap position (4) | -0.66 | 0.78 | -0.84 | 0.40 |
|  | **Treatment (YEOO)** | **-1.73** | **0.58** | **-2.97** | **0.003** |
|  | Location (Room) | -0.85 | 0.86 | -0.99 | 0.32 |
| “IY vs. water” in the semi-field setting | (Intercept) | -0.07 | 0.60 | -0.12 | 0.91 |
|  | Ovitrap position (2) | -0.51 | 0.79 | -0.64 | 0.52 |
|  | Ovitrap position (3) | -19.02 | 2521.71 | -0.01 | 0.99 |
|  | Ovitrap position (4) | -0.89 | 0.84 | -1.05 | 0.29 |
|  | Treatment (IY) | -1.14 | 0.70 | -1.64 | 0.10 |
|  | Location (Room) | -0.35 | 0.90 | -0.39 | 0.69 |

YEOO: yeast-encapsulated orange oil; IY: Inactivated yeast

- **Number of eggs**

When we analyse the data from egg number, wooden paddles present significant differences in “treatment” for all comparison with water (a higher number of eggs in ovitraps with water), and “position 2” in comparison “YEOO vs. water” under semi-field. The signals found in “treatment” were consistent in both presence/absence data and number of eggs. For solution, all significant signals found presence/absence data were also found in number of eggs. We also found two new signals associated with “oviposition position” but without any consistency among comparisons.

**Table C.** Results of the generalized linear models (negative binomial) of the number of eggs in wooden paddles inside ovitraps.

| **Dependent variable** | **Independent variable** | **Coefficient (ß)** | **Std. error** | **z-value** | **p-value** |
| --- | --- | --- | --- | --- | --- |
| “YEOO vs. water” in the laboratory | (Intercept) | 2.12 | 0.55 | 3.84 | 0.00 |
|  | Time (72H) | -0.51 | 0.46 | -1.09 | 0.28 |
|  | Ovitrap position (2) | 0.17 | 0.67 | 0.26 | 0.80 |
|  | Ovitrap position (3) | 0.93 | 0.66 | 1.41 | 0.16 |
|  | Ovitrap position (4) | 0.70 | 0.67 | 1.05 | 0.30 |
|  | **Treatment (YEOO)** | **-1.83** | **0.47** | **-3.86** | **< 0.001** |
| “YEOO vs. water” in the semi-field setting | (Intercept) | 3.24 | 0.73 | 4.45 | 0.00 |
|  | **Ovitrap position (2)** | **-3.91** | **1.14** | **-3.42** | **< 0.001** |
|  | Ovitrap position (3) | -0.91 | 0.95 | -0.96 | 0.34 |
|  | Ovitrap position (4) | -0.77 | 0.98 | -0.78 | 0.43 |
|  | **Treatment (YEOO)** | **-1.67** | **0.75** | **-2.22** | **0.03** |
|  | Location (Room) | 1.52 | 0.93 | 1.63 | 0.10 |
| “YEOO vs. IY” in the semi-field setting | (Intercept) | 1.89 | 0.49 | 3.82 | 0.00 |
|  | Ovitrap position (2) | -0.56 | 0.63 | -0.89 | 0.37 |
|  | Ovitrap position (3) | 0.24 | 0.63 | 0.38 | 0.71 |
|  | Ovitrap position (4) | 0.18 | 0.63 | 0.28 | 0.78 |
|  | Treatment (YEOO) | -0.22 | 0.45 | -0.49 | 0.62 |
|  | Location (Room) | 0.03 | 0.62 | 0.05 | 0.96 |
| “IY vs. water” in the semi-field setting | (Intercept) | 2.82 | 0.67 | 4.20 | 0.00 |
|  | Ovitrap position (2) | 0.43 | 0.86 | 0.49 | 0.62 |
|  | Ovitrap position (3) | 1.47 | 0.85 | 1.73 | 0.08 |
|  | Ovitrap position (4) | -0.23 | 0.88 | -0.26 | 0.80 |
|  | **Treatment (IY)** | **-3.17** | **0.61** | **-5.21** | **< 0.001** |
|  | Location (Room) | -0.62 | 0.80 | -0.77 | 0.44 |

YEOO: yeast-encapsulated orange oil; IY: Inactivated yeast

**Table D.** Results of the generalized linear models (negative binomial) of the number of eggs in solution inside ovitraps.

| **Dependent variable** | **Independent variable** | **Coefficient (ß)** | **Std. error** | **z-value** | **p-value** |
| --- | --- | --- | --- | --- | --- |
| “YEOO vs. water” in the laboratory | (Intercept) | 1.95 | 0.50 | 3.93 | 0.00 |
|  | Time (72H) | 0.69 | 0.43 | 1.62 | 0.11 |
|  | Ovitrap position (2) | 0.63 | 0.60 | -1.05 | 0.29 |
|  | Ovitrap position (3) | -0.77 | 0.60 | -1.29 | 0.20 |
|  | **Ovitrap position (4)** | **-1.52** | **0.63** | **-2.43** | **0.02** |
|  | **Treatment (YEOO)** | **-1.58** | **0.44** | **-3.60** | **< 0.001** |
| “YEOO vs. water” in the semi-field setting | (Intercept) | 0.46 | 0.69 | 0.67 | 0.50 |
|  | Ovitrap position (2) | -1.41 | 1.11 | -1.27 | 0.21 |
|  | **Ovitrap position (3)** | **-3.03** | **1.34** | **-2.27** | **0.02** |
|  | Ovitrap position (4) | -0.11 | 0.99 | -0.12 | 0.91 |
|  | Treatment (YEOO) | -1.73 | 0.88 | -1.96 | 0.05 |
|  | **Location (Room)** | **2.07** | **0.92** | **2.26** | **0.02** |
| “YEOO vs. IY” in the semi-field setting | (Intercept) | 0.58 | 0.52 | 1.13 | 0.26 |
|  | Ovitrap position (2) | 0.19 | 0.71 | 0.27 | 0.79 |
|  | Ovitrap position (3) | -0.20 | 0.71 | -0.28 | 0.78 |
|  | Ovitrap position (4) | -0.12 | 0.73 | -0.17 | 0.87 |
|  | **Treatment (YEOO)** | **-2.67** | **0.60** | **-4.44** | **< 0.001** |
|  | Location (Room) | 0.11 | 0.71 | 0.16 | 0.87 |
| “IY vs. water” in the semi-field setting | (Intercept) | **1.30** | **0.15** | **8.96** | **0.00** |
|  | Ovitrap position (2) | -0.06 | 0.19 | -0.34 | 0.73 |
|  | Ovitrap position (3) | -0.18 | 0.19 | -0.95 | 0.34 |
|  | Ovitrap position (4) | -0.08 | 0.19 | -0.44 | 0.66 |
|  | Treatment (IY) | -0.08 | 0.13 | -0.61 | 0.54 |
|  | Location (Room) | -0.03 | 0.18 | -0.18 | 0.86 |

YEOO: yeast-encapsulated orange oil; IY: Inactivated yeast
