# Supplementary figures and images for: The impact of yeast-encapsulated orange oil in Aedes aegypti oviposition
Source: PLoS One. 2024 May 14;19(5):e0301816. doi: 10.1371/journal.pone.0301816 (PMC11093346; doi:10.1371/journal.pone.0301816)

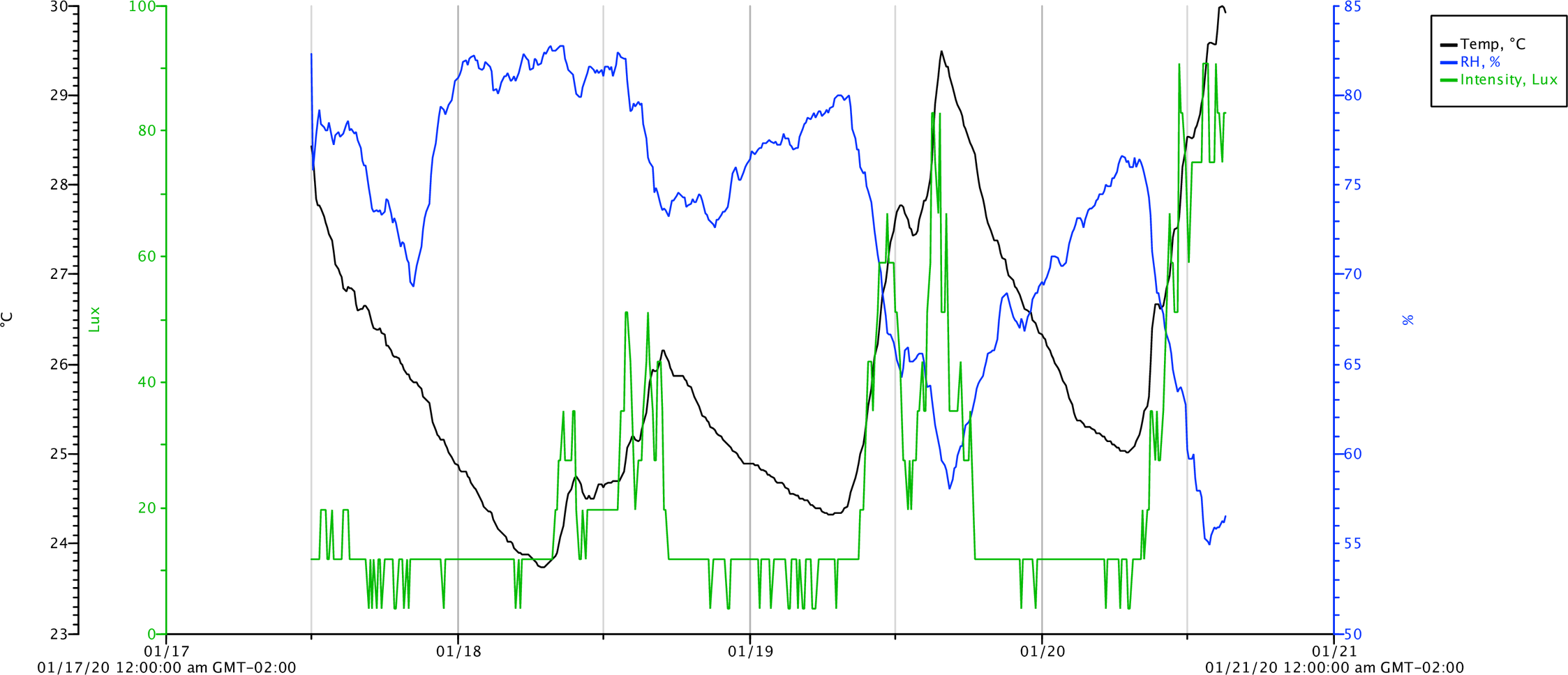

Supplement: S1 Fig — Location 1 is a courtyard protected from rain with natural light and air circulation. Black: temperature (°C), Blue: relative humidity (%), Green: light intensity (Lux). (TIF) [file pone.0301816.s002.tif]

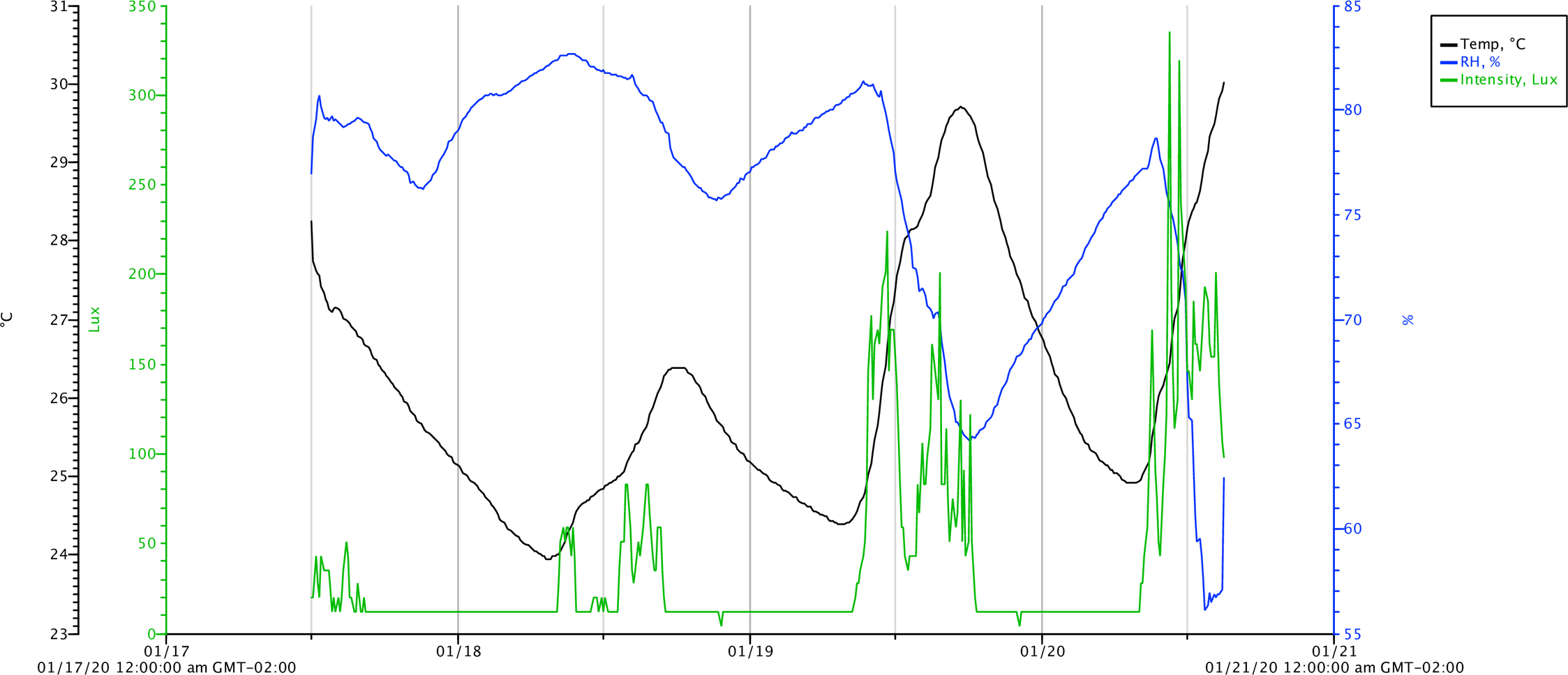

Supplement: S2 Fig — Location 2 is a room with a large window with natural light and restricted air circulation. Black: temperature (°C), Blue: relative humidity (%), Green: light intensity (Lux). (TIF) [file pone.0301816.s003.tif]

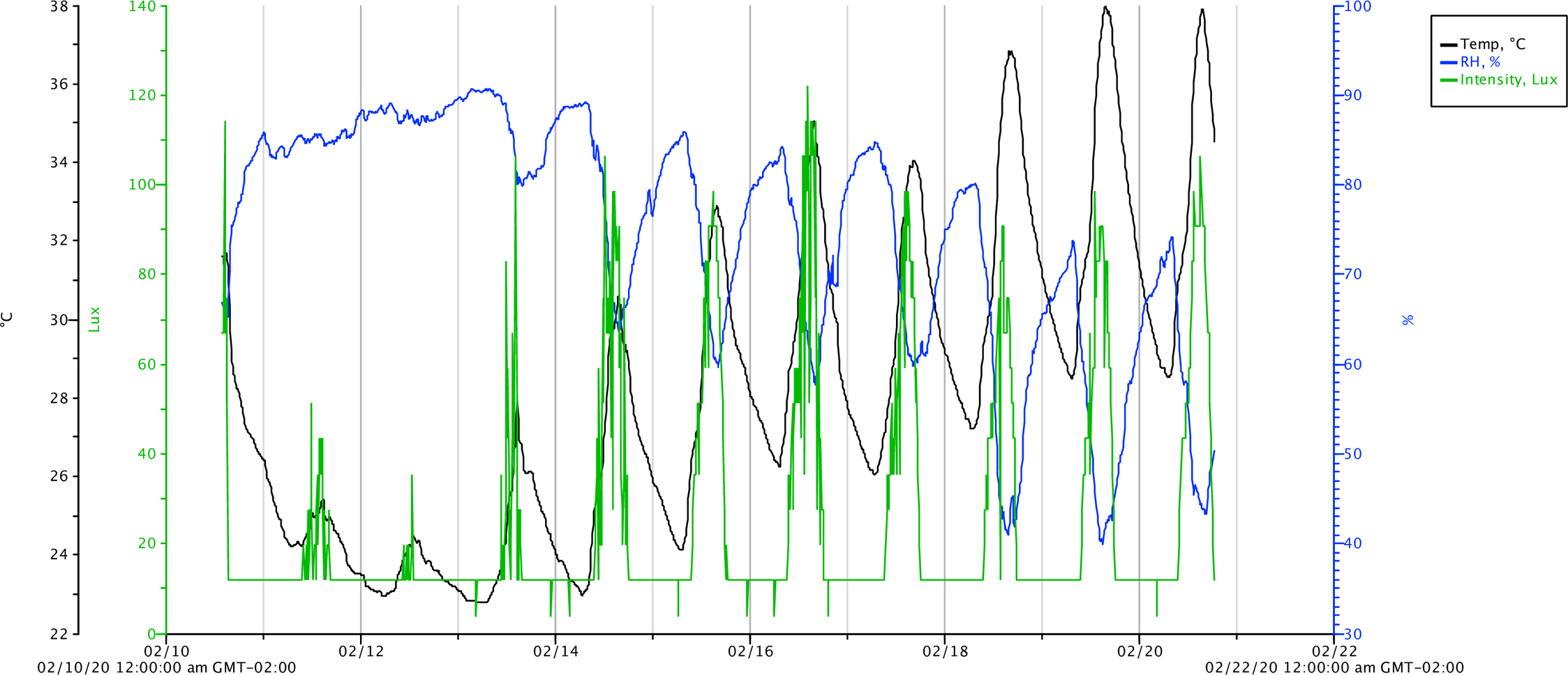

Supplement: S3 Fig — Location 1 is a courtyard protected from rain with natural light and air circulation. Black: temperature (°C), Blue: relative humidity (%), Green: light intensity (Lux). (TIF) [file pone.0301816.s004.tif]
