# Supplementary material for: The impact of yeast-encapsulated orange oil in Aedes aegypti oviposition
Source: PLoS One. 2024 May 14;19(5):e0301816. doi: 10.1371/journal.pone.0301816 (PMC11093346; doi:10.1371/journal.pone.0301816)
Supplement: S1 Table — Lab48h: 48h assays under laboratory conditions; Lab72h: 72h assays under laboratory conditions; Env72h: 72h assays at environmental conditions with natural light. (DOCX) [file pone.0301816.s005.docx]

**Table S1**. Descriptive statistic of mosquito eggs in five oviposition experiments

|  | Lab48h | | Lab72h | | Env72h | | Env72h | | Env72h | |
| --- | --- | --- | --- | --- | --- | --- | --- | --- | --- | --- |
|  | YEOO | water | YEOO | water | YEOO | water | YEOO | DY | water | DY |
| Number of values | 15 | 15 | 13 | 13 | 12 | 12 | 20 | 20 | 17 | 17 |
|  |  |  |  |  |  |  |  |  |  |  |
| Minimum | 0 | 2 | 0 | 1 | 0 | 0 | 0 | 0 | 0 | 0 |
| **25% Percentile** | **0.0** | **4.0** | **0.0** | **12.0** | **0.0** | **25.5** | **1.0** | **3.0** | **24.5** | **0.0** |
| **Median** | **1.0** | **22.0** | **2.0** | **29.0** | **0.0** | **32.5** | **6.0** | **20.5** | **31.0** | **0.0** |
| **75% Percentile** | **10.0** | **52.0** | **10.5** | **54.5** | **6.3** | **41.5** | **18.8** | **26.0** | **43.0** | **2.0** |
| Maximum | 39 | 77 | 17 | 87 | 38 | 57 | 47 | 46 | 63 | 43 |
| Range | 39 | 75 | 17 | 86 | 38 | 57 | 47 | 46 | 63 | 43 |
| Sum | 111 | 392 | 63 | 461 | 59 | 393 | 218 | 341 | 560 | 70 |

Lab48h: 48h assays under laboratory conditions; Lab72h: 72h assays under laboratory conditions; Env72h: 72h assays at environmental conditions with natural light.
